# High-Throughput *In Vitro* Gene Expression Profile to Screen of Natural Herbals for Breast Cancer Treatment

**DOI:** 10.3389/fonc.2021.684351

**Published:** 2021-08-13

**Authors:** Ling Kui, Qinghua Kong, Xiaonan Yang, Yunbing Pan, Zetan Xu, Shouling Wang, Jian Chen, Kunhua Wei, Xiaolei Zhou, Xingzhi Yang, Tingqin Wu, Anthati Mastan, Yao Liu, Jianhua Miao

**Affiliations:** ^1^Shenzhen Qianhai Shekou Free Trade Zone Hospital, Shenzhen, China; ^2^Dana-Farber Cancer Institute, Harvard Medical School, Boston, MA, United States; ^3^School of Pharmacy, Jiangsu University, Zhenjiang, China; ^4^State Key Laboratory of Phytochemistry and Plant Resources in West China, Kunming Institute of Botany, Chinese Academy of Sciences, Kunming, China; ^5^Guangxi Key Laboratory of Medicinal Resources Protection and Genetic Improvement, Guangxi Medicinal Botanical Garden, Nanning, China; ^6^Guangxi Engineering Research Center of Traditional Chinese Medicine (TCM) Resource Intelligent Creation, Guangxi Botanical Garden of Medicinal Plants, Nanning, China; ^7^Nowbio Biotechnology Company, Kunming, China; ^8^International Genome Center, Jiangsu University, Zhenjiang, China; ^9^Department of Cell Biology, Zhongshan School of Medicine, Sun Yat-Sen University, Guangzhou, China; ^10^Research Center, Microbial Technology Laboratory, Council of Scientific & Industrial Research (CSIR)-Central Institute of Medicinal and Aromatic Plants, Bangalore, India; ^11^Baoji High-tech Hospital , Baoji, China; ^12^School of Pharmacy, Guangxi Medical University, Nanning, China

**Keywords:** breast cancer, traditional Chinese medicine (TCM), high-throughput sequencing, transcriptome analysis, WGCNA

## Abstract

Breast cancer has surpassed lung cancer as the most commonly diagnosed cancer in women worldwide. Some therapeutic drugs and approaches could cause side effects and weaken the immune system. The combination of conventional therapies and traditional Chinese medicine (TCM) significantly improves treatment efficacy in breast cancer. However, the chemical composition and underlying anti-tumor mechanisms of TCM still need to be investigated. The primary aim of this study is to provide unique insights to screen the natural components for breast cancer therapy using high-throughput transcriptome analysis. Differentially expressed genes were identified based on two conditions: single samples and groups were classified according to their pharmaceutical effect. Subsequently, the sample treated with *E. cochinchinensis Lour.* generated the most significant DEGs set, including 1,459 DEGs, 805 upregulated and 654 downregulated. Similarly, group 3 treatment contained the most DEGs (414 DEGs, 311 upregulated and 103 downregulated). KEGG pathway analyses showed five significant pathways associated with the inflammatory and metastasis processes in cancer, which include the TNF, IL−17, NF-kappa B, MAPK signaling pathways, and transcriptional misregulation in cancer. Samples were classified into 13 groups based on their pharmaceutical effects. The results of the KEGG pathway analyses remained consistent with signal samples; group 3 presents a high significance. A total of 21 genes were significantly regulated in these five pathways, interestingly, IL6, TNFAIP3, and BRIC3 were enriched on at least two pathways, seven genes (FOSL1, S100A9, CXCL12, ID2, PRS6KA3, AREG, and DUSP6) have been reported as the target biomarkers and even the diagnostic tools in cancer therapy. In addition, weighted correlation network analysis (WGCNA) was used to identify 18 modules. Among them, blue and thistle2 were the most relevant modules. A total of 26 hub genes in blue and thistle2 modules were identified as the hub genes. In conclusion, we screened out three new TCM (*R. communis L.*, *E. cochinchinensis Lour.*, and *B. fruticosa*) that have the potential to develop natural drugs for breast cancer therapy, and obtained the therapeutic targets.

## Introduction

Among numerous diseases, cancer is the leading threat to modern human health. According to the GLOBOCAN 2020 estimates of cancer incidence and mortality produced by the International Agency for Research on Cancer/World Health Organization, there were 19.3 million new cancer cases and 10.0 million cancer deaths worldwide in 2020. The global cancer burden is expected to reach up to 28.4 million cases in 2040, a 47% rise from 2020 (1). Surgery, radiotherapy, and chemotherapy are the three major clinical measures to treat cancer effectively. Chemotherapy, however, produces serious adverse effects, and chemotherapy drugs also badly damage the immune function of the body. Breast cancer is one of the most commonly diagnosed malignant tumors among females. Female breast cancer has surpassed lung cancer as the most commonly diagnosed cancer, with an estimated 2.3 million new cases (11.7%). Death rates of female breast cancer were considerably higher in developing countries than developed ones (15.0 *vs* 12.8 per 100,000) (1). Currently, the main treatments for breast cancer include surgery and postoperative chemotherapy, but most chemotherapy drugs kill both cancer and normal cells (2–5). Their side effects of chemotherapies somewhat impact the quality of life of the patients.

On the other hand, traditional Chinese medicine (TCM) relieves adverse effects and enhances the efficacy of drugs (6–8). Clinical trials have shown that the combination of modern medical methods and TCM in the cancer treatment significantly reduces the toxicity and side effects of both radiotherapy and chemotherapy, decreases surgical complications, prolongs patient survival, and effectively improves their life quality (3, 9). Hence, owing to its high efficiency, low cost, and minimal side effects, plant-based medicinal therapies are very important for the treatment of cancer (10). TCM is also found to have cytoprotective properties during combinational chemotherapy without hindering the anti-cancer activity of conventional drugs (11). An increasing number of medical practitioners have focused on finding the active anti-cancer ingredients from TCM and reporting their anti-cancer mechanisms (12, 13). Therefore, research to develop new drugs for breast cancer treatment has focused on finding natural ingredients with low toxicity and high efficiency from TCM (14, 15).

Guangxi Botanical Garden of Medicinal Plants, Nanning, China has collected and conserved a number of plant species belonging to different families including Euphorbiaceae (*Ricinus*, *Mallotus*, *Euphorbia*, etc.), Araliaceae (*Panax*), Asteraceae (*Acmella*, *Arctium*, *Gynura*, etc.), Fabaceae, Malvaceae, Solanaceae, and so on. Almost 74 kinds of fractions (**Supplemental Table 1**) have significant medical effects, including promoting blood circulation and removing stasis; heat-Clearing; Rheumatism treatment; hemostatic; tonic; asthmatics, expectorants and antitussives; water-disinhibiting and damp-percolating; Qi-regulating; heat-clearing astringent; detoxifying, analgesia, antipruritic, etc. In previous studies, some of the plant materials with potential anti-cancer effects have been analyzed, such as *Excoecaria cochinchinensis Lour.*, *Breynia fruticosa*, *Salvia miltiorrhiza Bunge*, *Gynura procumbens*, *Euphorbia hirta*, *Forsythia suspensa*, *Senecio scandens*, *Polygonum perfoliatum*, *Malva verticillata* var. *crispa*, *Mallotus apelta*, *Pteris semipinnata*, *Acmella paniculate*, *Aristolochia tagala*, *Schisandra chinensis*, *Gelsemium elegans*, *Ricinus communis L*., *Ligustrum confusum*, etc. (10, 16–20). Most of them have been found to contain anti-cancer compounds like terpenoid, flavonoid, alkaloid, quercetin, epigallocatechin-3-gallate (EGCG), epicatechin, oleanolic acid, ursolic acid, and tanshinone, which are involved in multiple important pathways in breast cancer such as epithelial to mesenchymal transition (EMT), TGF-ß, PTEN/PI3K/Akt, NF-kappa B (NF-κB), MAPK, p53 signaling pathway, and Wnt/β-catenin pathways (21–24).

However, the anti-cancer compounds and molecular mechanisms of TCM are still unclear, therefore, it is still necessary to further investigate their actual effectiveness and active compounds. Previous studies have reported that the chemical compounds with anti-cancer activities, such as Cisplatin, Paclitaxel, Docetaxel, Doxorubicin, Gefitinib, Cabozantinib, and so on (5, 21, 25–28), can be used in a combination chemotherapy for breast cancer. Undoubtedly, high-throughput screening is an effective and systematic approach to analyze TCM-mediated gene expression modifications in breast cancer cells. The underlying anti-cancer mechanisms of conventional drugs and traditional medicines can be analyzed on the basis of gene expression and pathway enrichment variations in different treatments. Afterwards, the pharmaceutical effects and effective components are comprehensively considered to infer the potential TCM fractions for breast cancer therapy. Moreover, this may help to identify the molecular mechanisms and anti-cancer compounds involved to help develop natural cancer treatment drugs.

Transcriptome analysis reveals the full information about differentially expressed genes in different treatments from specific cell types or tissues (29, 30). The regulatory mechanisms of cancer cells are associated with various signaling pathways and their differentially expressed genes. Comparative transcriptome analysis can be used to assess the interactions between drugs and cellular activities, expression levels of functional genes of different cell lines, and changes in the activity of cellular regulatory mechanisms and signaling pathways (31–35). The data from this analysis can be used to characterize anti-cancer compounds and drug development. Thus, these findings on drug-cell interactions provide accurate scientific insights for the development of alternative chemotherapies (33).

Weighted gene co-expression network analysis (WGCNA) is a bioinformatics data mining method that has been used to explore the relationships among the gene modules of various cancer cell lines (36, 37). The modules of co-expressing genes are found to maintain a consistent phenotype-independent expression relationship and they may co-regulate and share common biological functions (37). Expression alterations and intrinsic properties of gene sets, correlation between gene modules, phenotype-correlated modules, candidate biomarker genes, and targets of therapeutic drugs can be analyzed through WGCNA (38). In previous studies, WGCNA was used to analyze biomarkers and targets of various diseases like schizophrenia, Alzheimer’s disease, sickle cell disease, and breast cancer (39–42). WGCNA is a systematic biological method which has been used in cancer studies including bladder cancer, clear cell renal cell carcinoma (ccRCC), Non-Small-Cell Lung Cancer (NSCLC), Acute myeloid leukemia (AML), and Pancreatic ductal adenocarcinoma (PDAC) (38, 43–46). Tang et al. applied WGCNA to construct a gene co-expression network and to explore and measure the relationships between genes, modules, and clinical traits, and they identified five hub genes, CCNB2, FBXO5, KIF4A, MCM10, and TPX2, which are found to be associated with the progression and poor prognosis of breast cancer (47). Liu et al. used 22 human osteosarcoma cell lines to construct gene co‐expression modules to predict the groups of candidate genes responsible for the pathogenesis of osteosarcoma, and they identified seven co‐expression modules containing 2,228 differentially expressed genes (36). Using WGCNA, Zhai et al. constructed the co−expression network of DEGs and identified the recurrence-associated genes (SERP2, EFEMP2, FBN1, SPARC, and LINC0219) in colon cancer to prevent tumor recurrence (48). Lin et al. used weighted gene co-expression network analysis to find the genes responsible for tumor progression and CD8+ T cell infiltration in ccRCC, finding that CCL5 is a potential biomarker and therapeutic target to treat ccRCC (49). Moreover, the pathway-related modules and hub genes have also been identified in other diseases like schizophrenia (50) and intracranial aneurysm (51). WGCNA has also been used to analyze the differentially expressed genes and signaling pathways associated with inflammatory/immune processes throughout time points after a burn, and to identify key genes related to pathologic changes after a severe burn (52).

This study used high-throughput transcriptome sequencing to provide the transcriptome data of breast cancer cell line distilled with the aforesaid 74 TCM and 10 chemical compounds (positive control). High-quality data sets are obtained from transcriptome assembly and comparative analysis, and, subsequently, the key genes responsible for the pro-inflammatory effects and metastasis of breast cancer were identified and validated by differential expression and WGCNA. By comparing the changes in the gene expression among the cancer-related pathway of cell lines treated with TCM or chemical compounds with a known anti-cancer effect, we identified the potential TCM with anti-cancer effects. The primary aim of this study is to establish a high-throughput method to screen both the natural anti-cancer compounds and their target genes and to provide new insights to develop alternative anti-cancer chemotherapies for breast cancer.

## Materials and Methods

### Preparation of Medicinal Plant Extracts

A total of 74 medicinal plants samples were obtained from Guangxi Botanical Garden of Medicinal Plants. Dried plant materials were ground into fine powder using a mortar and pestle. We marked the samples with different serial numbers according to their extraction methods: The samples named “W” refer to the plant materials that were boiled by hot water then freeze dried; ‘GX’ refers to the medicinal plants that were extracted using 60% ethanol for 2 h. The samples were firstly vacuum concentrated and loaded on a macroreticular resin column, the product of interest was then eluted by water. Finally, the eluted fractions were concentrated by vacuum evaporation and the completely dried samples were used for the drug preparation. For the dried fruit of *Myristica fragrans*, we applied CO_2_ supercritical extraction method, and named the sample as ‘C’. ‘S’ refers to the remaining plant materials which were firstly extracted by petroleum ether for 2 h and filtered; the remaining residue was then extracted by ethyl acetate for another 2 h; the extracts were finally vacuum concentrated to dry powder for the drug preparation. More description on the samples are shown in **Supplemental Table 1**. While, 10 conventional anti-cancer drugs (positive control) were purchased from various companies (**Table 1**) and all the plant samples and drugs were dissolved in DMSO for anti-cancer activity, marked as the fractions and positive control, respectively.

**Table 1:** The list of positive control.

Drug	Source (company)
Sorafenib	Beijing solarbio science & technology Co., Ltd
Cisplatin	Beijing solarbio science & technology Co., Ltd
Lenvatinib	Shanghai Topscience Bio-Technology Co., Ltd
Cabozantinib	Shanghai yuanye Bio-Technology Co., Ltd
Doxorubicin hydrochloride	Shanghai yuanye Bio-Technology Co., Ltd
Gefitinib	Shanghai Macklin Biological Co., Ltd
Paclitaxel	Shanghai Macklin Biological Co., Ltd
Docetaxel	Shanghai Macklin Biological Co., Ltd
Viborelbine	Shanghai Aladdin Reagent Co., Ltd
Gemcitabine	Beijing OKA Bio-Technology Co., Ltd

### Cell Culture and Drug Preparation

MCF-7 cell line was procured from Shanghai Cell Bank of the Chinese Academy of Sciences, and the cell line was cultured in a DMEM medium containing 10% fetal bovine serum and placed in a cell incubator with 5% CO_2_ at 37°C. When the cell growth density reached 80–90%, trypsin (0.125%) was used to subculture the cells on a complete culture media. The anti-cancer activity of the test samples was screened on the MCF-7 cancer cells during a logarithmic growth phase, a total of 74 TCM and 10 conventional anti-cancer drugs were used (**Supplemental Tables 1**, **8** and **Table 1**). The samples treated with 10 conventional anti-cancer drugs were considered as the positive control and the 7 samples without any treatment were kept as the negative control. The fractions mainly came from Leguminosae, Compositae, Araliaceae, Euphorbiaceae, Rutaceae, etc., all of which were verified to have anti-tumor effects through preliminary investigation. The positive controls were purchased from the companies (**Table 1**).

The cell lines were treated with the fractions and positive control. Initial screening was carried out at a concentration of 100 μg/ml, with a final volume of 200 μl per well, and the cell survival rate after 24 h of drug treatment was calculated. If the cell survival rate was more than 80%, 6-well plate screening was carried out using the same concentration. If the survival rate was less than 80%, the 96-well plate screening was carried out at a reduced concentration. The fractions were screened with a gradient of two times. With reference to the cell survival rate observed in the first screening, the 6-well plate screening would be carried out at the concentration at which the cell survival rate reached about 80% (**Supplemental Figure 1**). This concentration was recorded as the final administration concentration, at which the cell growth conditions would be observed under a microscope.

### RNA Quality Control, Library Preparation, and Sequencing

The total RNA of the cell lines was extracted using the FastPure Cell/Tissue Total RNA Isolation Mini Kit (Vazyme, Nanjing, China), and identified OD260/280 = 1.8~2.0 using the NanoDrop 2000 and Agilent 2100 Bioanalyzer (Agilent, USA). The RIN (RNA Integrity Number) values of all total RNA samples were above 8, then their concentration was accurately quantified by Q-bit. The MGIEasy kit was used for mRNA library preparation. Total RNA samples were used for the preparation of cDNA-libraries according to the standard BGISEQ protocol, and insert size is 200–300 bp. Paired-end sequencing with 100 bp read length were sequenced using the BGISEQ-500 instrument and BGISEQ-500RS high-throughput sequencing kit (**Supplemental Figure 1**).

### RNA-Seq Clean Data Preparation and Quality Checking

The resulting sequencing data were collected in FASTA format for further analysis. FastQC was used to detect and provide evidence of problematic sequencing data (53). Post-sequencing quality assessment can identify problematic libraries: such as those with low quality base-call scores, a shift from the expected GC-content, or overrepresented adapter sequences. FastQC can help ensure that high-quality data is provided to the initial spliced alignment step. Subsequently, the read sequences were subjected to adapter trimming and quality filtering using the Trimmomatic software (v.0.36), which is included in the Trinity package (54, 55). More details about quality checking are shown in **Supplemental Table 2** and the generated clean data has been uploaded to the NCBI Sequence Read Archive (SRA). In order to take multiple correlation analysis and screen the potential natural components for breast cancer therapy, the samples were classified into 13 groups based on their pharmaceutical effects (**Supplemental Table 1**), which included promoting blood circulation and removing stasis; heat-clearing; Rheumatism treatment, hemostatic; tonic; asthmatics, expectorants and antitussives; water-disinhibiting and damp-percolating; Qi-regulating; heat-clearing astringent; detoxifying, analgesia, and antipruritic. Samples treated with TCM fractions were labeled as groups 1–10; group 11 was composed of the samples that were treated with unclassified fractions. While, the positive and negative controls were marked as groups 12 and 13, respectively.

### General Analyzing of RNA-Seq: Alignment, Transcript Assembly, Differential Expression, and KEGG Pathway Analysis

After quality control, the RNA-seq analysis generally requires four steps: aligning the reads to the reference; assembling the alignments on the alignment into a full-length transcript; quantitative expression of genes and transcripts; and calculation of the differential expression of all genes under different experimental conditions. The “New Tuxedo” package including HISAT, StringTie, and Ballgown was used for this process (**Supplemental Figure 2**). Our pipeline begins with raw RNA-seq reads generated by the BGISEQ-500 instrument and produces several useful outputs, including the lists of genes, transcripts, and expression levels for each sample, tables showing the differentially expressed genes in different conditions, and accompanying measures of statistical significance.

In this pipeline, HISAT (56) was used to align the RNA-seq reads to the genome. It compares and finds the variable splicing sites faster and consumes less memory than Tophat2 (57). StringTie (58) is responsible for assembling transcripts and constructing isoforms to estimate the gene expression. First, it receives the alignments from Hisat for transcript splicing. Each data set is independent. During the splicing process, the expression of each gene and each isoform is estimated. After the splicing, all the spliced sequences are merged. This step is necessary because the transcripts in some samples are only partially covered by reads, with the result that only the covered areas are spliced. This error can be reduced by “merge”. The merged transcript is returned to StringTie again to calculate the transcript abundance. It uses the same algorithm as the initial splicing, but if the abundance of the transcript does not change during the merge process, yet the structure does, the reads also need to be adjusted accordingly. StringTie also provides a read-count for each transcript, and this data will be passed to Ballgown (59), which uses the results of StringTie splicing to calculate the gene expression, then obtain the FPKM (Fragments Per Kilobase Million) results.

Differential expression analysis was performed using the DESeq2 Wrapper included in the TBtools, a toolkit for biologists integrating various biological data-handling tools (60). The selection criteria for the differential expressed genes (DEGs) were strengthened with a threshold of padjust ≤ 0.05 and |log2FC| ≥ 1 (61), and the distribution of each gene was illustrated by a Volcano plot. Differentially expressed genes were identified based on two conditions: 1) single sample distilled with 74 TCM fractions compared to CK, aiming to investigate the anti-tumor mechanism of each fraction through enriched pathways and screen the potential medicinal composition; and 2) groups classified according to their pharmaceutical effect, comparing the fractions groups with the positive control (including chemotherapy drugs) and negative control, to obtain the groups with an anti-tumor effect. The differential expression was analyzed in both single samples and groups, so that screening the TCM fractions with significant effect using the high-throughput and systematic methods.

Finally, the DEGs were imported to analyze the gene ontology and KEGG pathway by the R package clusterProfiler and org.Hs.eg.db data set, with the parameters set as pvalue Cutoff = 0.05, pAdjustMethod = “BH”, and qvalueCutoff = 0.1. Based on the KEGG results, the candidate pathways associated with the inflammatory and metastasis processes in cancer, as well as the genes enriched on these candidate pathways, were selected from the top 10 most significant pathways. Subsequently, TBtools was used to generate a heatmap of these genes to visualize their expression; the common genes were screened according to their significantly differential expression. The analysis steps, software, and main scripts in our pipeline are listed in **Supplemental Table 3**.

### Weighted Gene Co-Expression Network Analysis (WGCNA)

The modified WGCNA pipeline used in this study is shown in **Supplemental Figure 2** and **Supplemental Table 3**. Construction of weighted co-expressed networks and identification of co-expression modules were carried out using the WGCNA package in R with the default parameters. Importing the log2 normalized expression data, a sample information table was created in 13 groups. In order to ensure a scale-free network, the power of β = 11 (scale free R2 = 0.8) was selected as the soft-thresholding and MEDissThres was set as 0.25 to merge similar modules. After that, a functional enrichment analysis was performed to examine the enrichment of the modules. The genes that were included in all 18 modules were extracted and then imported to the KEGG pathway analysis with the parameters of pvalueCutoff = 0.05, pAdjustMethod = “BH”, and qvalueCutoff = 0.1. Checking the pathways and comparing them with the differential expression analysis results, common pathways associated with cancer were selected to be taken into consideration as the key pathways. Finally, screened edges by the criteria with weight value > 0.035 and weight value > 0.24 for thistle2 module and blue module, respectively, then inputted them into Cytoscape to (v3.8.1) visualize the co-expression network and identify the nodes and hub genes (62). Cytoscape (v3.8.1) software was downloaded from the website (https://cytoscape.org/) and three models were chosen including “source node”, “target node”, and “edge attribute” with default parameters (63). These genes may play an important role in the tumorigenesis process, which can be selected as the targets in future research on cancer therapy.

## Results

### Data Pre-Processing and Differential Expressed Genes (DEGs) Screening

**Figure 1:**
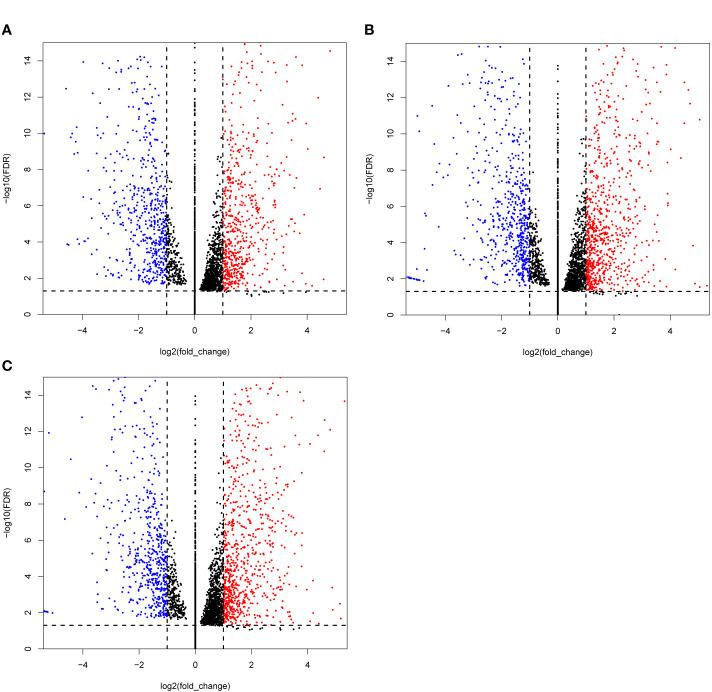
Volcano plots of the differentially expressed genes (DEGs) MCF-7 cell lines were treated with three candidate medicines. **(A)** *R. communis L.*, **(B)** *E. cochinchinensis Lour*., **(C)** *B*. *fruticose*. The horizontal line at |log2FC| = 1; vertical line at false discovery rate (FDR) = 0.05.

In this study, 99 cDNA libraries were sequenced and about 806.33 Gb raw data were produced, consisting of 8,063,347,526 reads with an average read length of 100 bp (**Supplemental Tables 3**, **4**). Clean sequencing reads (772.78 Gb and 7,759,550,456 reads) are available at the NCBI Sequence Read Archive. After quality control, the obtained sequencing reads were mapped to the reference genome using STAR and sorted using the Samtools. Subsequently, the gene expression (FPKM, Fragments Per Kilobase Million) was generated using the RSEM. Differential expression analysis was performed by the DESeq2 Wrapper included in the TBTools, the selection criteria for differential expressed genes (DEGs) were strengthened with a threshold of padjust ≤ 0.05 and |log2FC| ≥ 1 (61). For the DEGs analysis, the test samples were taken under two conditions: 1) single treat sample compared to CK; and (2) TCM groups compared to the CK group. Condition 1 obtained 91 DEGs sets. The set that contained the most DEGs is Treat58 *vs* CK, including 1,459 DEGs, 805 upregulated and 654 downregulated. We listed the top 10 sets shown in **Table 2**, and selected the DEGs set of three TCM candidates (*R. communis L*., *E. cochinchinensisLour*., *B. fruticosa*) as illustrated by a volcano plot (**Figure 1**). Similarly, condition 2 obtained 12 DEGs sets, group 3 *vs* CK contained the most DEGs, including 414 DEGs, 311 upregulated and 103 downregulated. The detailed DEGs information is shown in **Supplemental Table 5**.

**Table 2:** Differential gene expression of the top 10 sets in condition 1.

Compare	Significant	Upregulated	Downregulated	Sample name	Note
Treat58_*vs*_CK	1,459	805	654	*E. cochinchinensis Lour.*	Candidate
Treat73_*vs*_CK	1,410	803	607	*B. fruticosa*	Candidate
Treat15_*vs*_CK	1,371	735	636	*S. scandens*	
Treat16_*vs*_CK	1,321	583	738	*G. elegans*	
Treat24_*vs*_CK	1,316	647	669	*M. azedarach*	
Treat27_*vs*_CK	1,265	622	643	*R. communis L.*	Candidate
Treat21_*vs*_CK	1,047	492	555	*M. barbatus*	
Treat88_*vs*_CK	767	470	297	*P. reticulatus Poir.* var. *glaberMuell.-Age.*	
Treat7_*vs*_CK	747	251	496	*A. lappa*	
Treat28_*vs*_CK	714	333	381	*M. verticillata* var. *crispa*	

### KEGG Pathway Analysis and Candidate Drugs Screening

**Figure 2:**
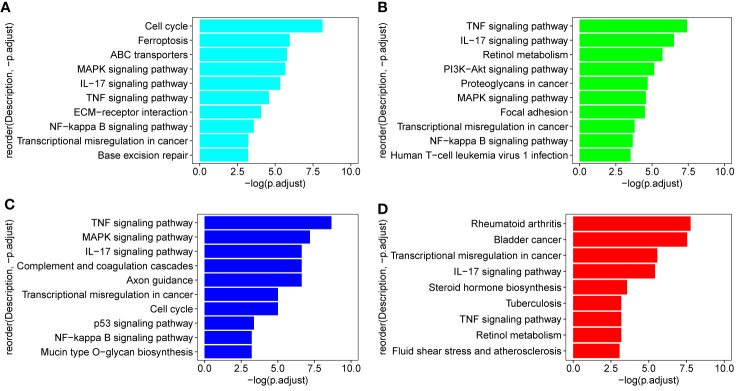
KEGG pathways in three candidate drugs and group 3. **(A–C)** Three TCM (*R. communis L*., *E*. *cochinchinensis Lour*., *B*. *fruticosa*) which were enriched on all five pathways: TNF signaling pathway, IL−17 signaling pathway, NF-kappa B signaling pathway, Transcriptional misregulation in cancer, and MAPK signaling pathway; **(D)** group 3 includes three important pathways: IL−17 signaling pathway and TNF signaling pathway participated in the pro-inflammatory process; Transcriptional misregulation in cancer is important for cancer metastasis process.

**Figure 3:**
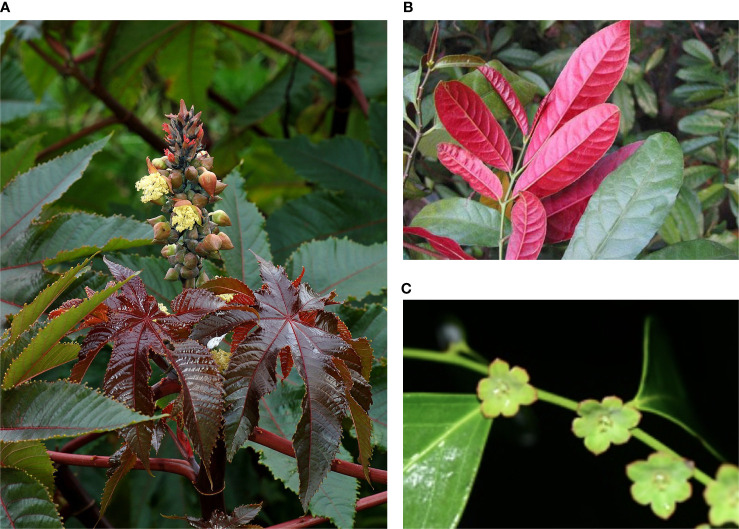
Plant morphology of the three TCM. **(A)** *Ricinus communis* L (Source: Wikipedia), **(B)** *Excoecaria cochinchinensis* Lour, and **(C)** *Breynia fruticosa* (Source: www.iplant.cn).

In order to identify the biologic pathways, networks, and functional categories of the differentially expressed genes, we used the R package clusterProfiler and org.Hs.eg.db data set to complete the Gene Ontology and KEGG pathway analysis, the parameters are set as pvalueCutoff = 0.05, pAdjustMethod = “BH”, and qvalueCutoff = 0.1. In order to investigate which TCM fractions can be potentially applied for cancer treatment, the samples were classified into 13 groups according to their pharmaceutical effect (**Supplemental Table 1**). KEGG analyses showed that the pathways which are involved in the cell cycle, cell proliferation, oncogenic transformation, pro-inflammatory effect, and cancer metastasis process are significantly present in groups 1–10 and 12. Previous research has shown that biological processes with a pro-inflammatory effect are important for cancer tumorigenesis, especially breast cancer (64–68). Transcriptional regulation is an important biological process in cancer metastasis (69). Fortunately, group 3 includes three important pathways involved in the pro-inflammatory and metastasis process, IL−17 signaling pathway, TNF signaling pathway, and Transcriptional misregulation in cancer (**Figure 2D**). KEGG analysis results of signal samples indicated that the five pathways were associated with the biological processes of the pro-inflammatory effects and that the metastasis process are significantly enriched, including the TNF signaling pathway, IL−17 signaling pathway, NF-kappa B signaling pathway, Transcriptional misregulation in cancer, and MAPK signaling pathway (**Figures 2A–C**). Among all, the three plant samples (*R. communis L*., *E. cochinchinensis* Lour., and *B. fruticosa*) significantly regulated the expression of key genes in the five signaling pathways, which were screened as the effective TCM candidates for future breast cancer therapy (**Figures 2A–C**; **Figure 3**). Interestingly, all three of these plants belong to the Euphorbiaceae family, indicating that the plant species of Euphorbiaceae may play an important role in breast cancer treatment. Among them, *E. cochinchinensis Lour*. and *B. fruticosa* belong to the same group (Group 3) with the pharmaceutical effect of Rheumatism treatment. *R. communis L*. was classified as a purgative drug. They were all known to have anti-inflammatory ingredients and anti-cancer effect in previous research. The detailed pathway results of other TCM are shown in **Supplemental Table 6**.

It is believed that breast cancer, especially HER2+-subtype and triple-negative breast cancer (TNBC), are associated with local inflammation. Biological processes with pro-inflammatory effects may play a potential role in breast cancer (64). Cancer is known to be regulated by inflammation that recruits resident or circulating immune cells at several stages of its development to modulate the tumor microenvironment (67, 68). Inflammation is constantly present in cancers. Except the Transcriptional misregulation in cancer associated with the cancer metastasis process, the other four candidate pathways and included factors were proved to be proinflammatory cytokines and important in tumorigenesis in previous research. Interestingly, they are interrelated to some extent. It is reported that TNF can stimulate apoptosis in certain pathological situations, whereas the major biochemical functions of TNF are realized by inducing NF-kappa B and MAP pro-survival kinase activities (70, 71). Activating NF-kappa B would enhance the expression of several inflammatory cytokine genes, including TNF-α, IL-6, and IL-8 (72). IL-17 signaling pathway is primarily activated by the NF-kappa B and MAPK pathways, and IL-17-family activated pro-inflammatory factor NF-kappa B in innate immune signaling. Numerous studies have shown that IL-17A activates various MAPKs, and the MAPK pathway has an important role in regulating the expression of IL-17A-induced genes through the control of mRNA transcript stability (67, 73–75). Similarly, Transcriptional misregulation also interacted with other pathways, misregulation of the immune response transcriptional regulator NF-kappa B has been linked to inflammation in cancer and the transcription factor-controlled genes involved in inflammation, and it is chronically active in cancer inflammation process (76).

### Expression and Regulation of Top Genes Enriched on These Five Pathways in Three Candidate TCM

**Figure 4:**
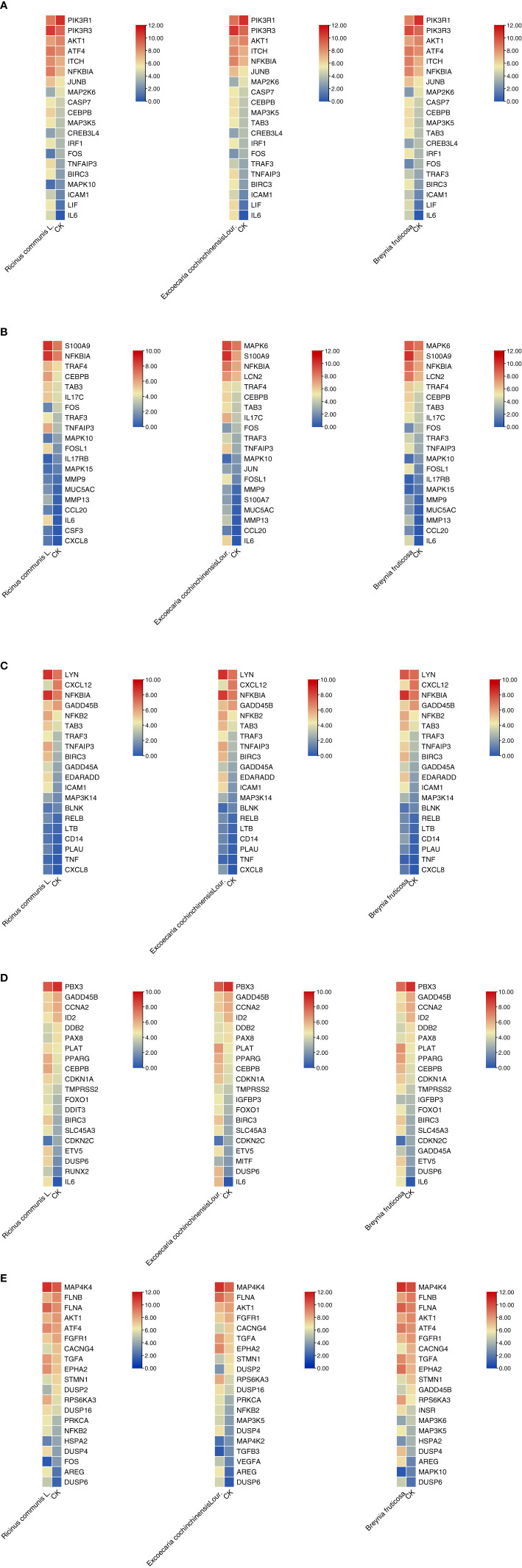
Expression and regulation of the top genes enriched on these five pathways in three candidate TCM. **(A)** TNF signaling pathway; **(B)** IL−17 signaling pathway; **(C)** NF-kappa B signaling pathway; **(D)** Transcriptional misregulation in cancer pathway; **(E)** MAPK signaling pathway. R: *R. communis L*.; E: *E*. *cochinchinensis Lour*.; B: *B*. *fruticose*; CK: the negative control.

**Figure 5:**
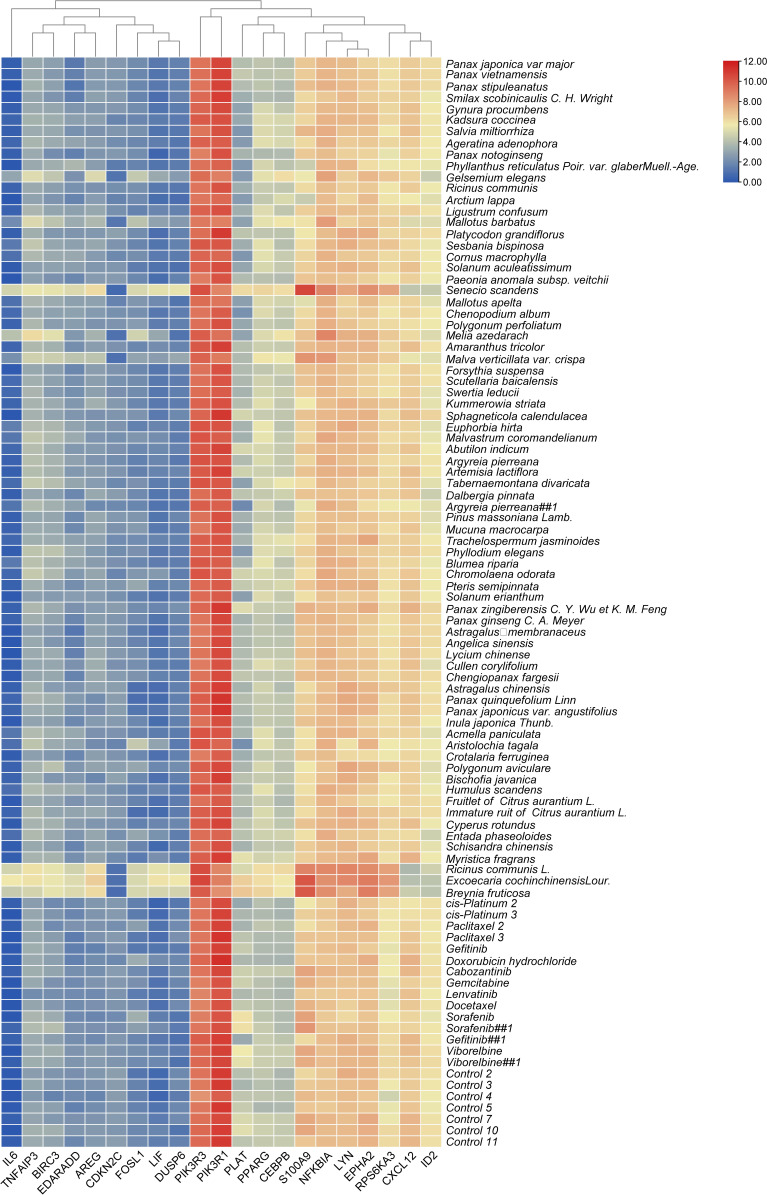
The expression levels of common genes in different treatments.

The top 20 genes with the greatest upregulation and downregulation enriched in these five pathways in three candidate TCM are shown in **Figure 4**. The commonly significantly regulated genes in three candidate TCM associated with TNF signaling pathway are IL6, LIF, PIK3R1, and PIK3R3; among which, PIK3R1 is downregulated and the other three are upregulated. In the IL−17 signaling pathway, IL6, FOSL1, TNFAIP3, and S100A9 are commonly significantly upregulated in three candidate TCM. CXCL12 is downregulated and LYN, NFKBIA, TNFAIP3, BRIC3, and EDARADD are upregulated in three candidate TCM and enriched on the NF-kappa B signaling pathway. Besides, CDKN2C and ID2 are downregulated while IL6, BIRC3, PLAT, PPARG, and CEBPB are upregulated in three candidate TCM associated with Transcriptional misregulation in the cancer pathway. In the MAPK signaling pathway, RPS6KA3, AREG, DUSP6, and EPHA2 are all upregulated in three candidate TCM. **Table 3** summarizes the common genes in three candidate TCM associated with the five pathways, and **Figure 5** shows the expression of common genes in all the samples.

**Table 3:** The summary of the common genes of the five pathways in cancer cells treated with three TCM candidates.

Pathway	Common genes in medicine	Regulate
TNF signaling pathway	*IL6*	upregulated
	*LIF*	upregulated
	*PIK3R1*	downregulated
	*PIK3R3*	upregulated
IL−17 signaling pathway	*IL6*	upregulated
	*FOSL1*	upregulated
	*TNFAIP3*	upregulated
	*S100A9*	upregulated
NF-kappa B signaling pathway	*CXCL12*	downregulated
	*LYN*	upregulated
	*NFKBIA*	upregulated
	*TNFAIP3*	upregulated
	*BRIC3*	upregulated
	*EDARADD*	upregulated
Transcriptional misregulation in cancer	*IL6*	upregulated
	*BIRC3*	upregulated
	*CDKN2C*	downregulated
	*ID2*	downregulated
	*PLAT*	upregulated
	*PPARG*	upregulated
	*CEBPB*	upregulated
MAPK signaling pathway	*RPS6KA3*	upregulated
	*AREG*	upregulated
	*DUSP6*	upregulated
	*EPHA2*	upregulated

It is worth noting that some of the common genes have been demonstrated to play an important role in the inflammatory or metastasis processes in cancers, especially in breast cancer. All the common genes analyzed were differentially expressed in the different TCM treatments. In particular, IL6, S100A9, RPS6KA3, AREG, ROSL1, LIF, and DUSP6 were significantly upregulated (**Figure 5**). Moreover, some genes such as IL-6, PIK3R1, LYN, TNFAIP3, S100A9, CXCL12, and ARGE exhibited the same level of expressions across the different treatments. Subsequently, seven common genes, including FOSL1, S100A9, CXCL12, ID2, PRS6KA3, AREG, and DUSP6, have been used as the target biomarkers for cancer diagnosis and therapy. The results indicated that the TCM candidates significantly inhibited the breast tumorigenesis by regulating various common genes of different signaling pathways. Overall, this study has inferred that our TCM candidates probably possessed the significant curative effects for breast cancer. Subsequent studies will consider the identification of effective compounds and functional investigation of common genes.

### Construction of Weighted Gene Co-Expression Network and Identification of Significant Modules Associated With Cancer

**Figure 6:**
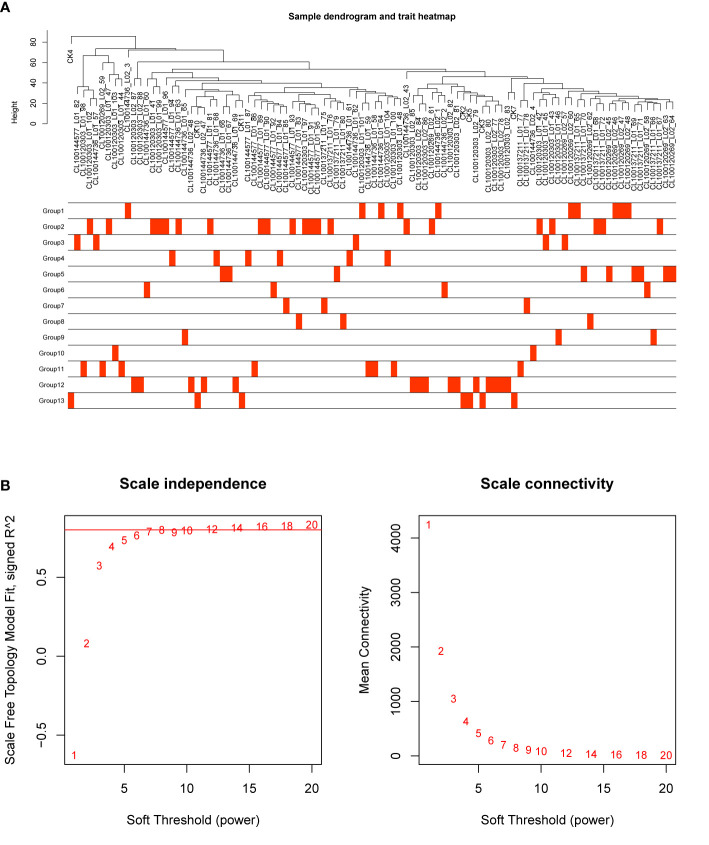
Sample dendrogram and estimation of soft-thresholding values. **(A)** Sample dendrogram and trait heatmap. The cut height was set to 80. The traits of 13 groups were classified according to their pharmaceutical effect. Group 12 represents the positive control and group 13 represents the negative control. **(B)** Scale independence and mean connectivity of various soft-thresholding values (β), power of β = 11 (scale free R2 = 0.8).

**Figure 7:**
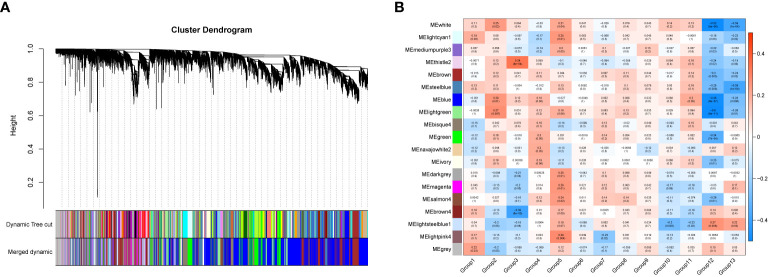
The gene set enrichment analysis and module identification. **(A)** Dendrogram of all filtered gene sets enriched according to a dissimilarity measure (1- TOM) and the cluster module colors. The first set of modules was obtained with the Dynamic Tree Cut algorithm and the correlated modules were merged together. Each branch in the figure represents one gene and every color below represents one co-expression module. **(B)** Heatmap of the correlation between the pharmaceutical effect of TCM fractions and MEs in breast cancer. The darker the color of the module, the more significant the relationship.

**Figure 8:**
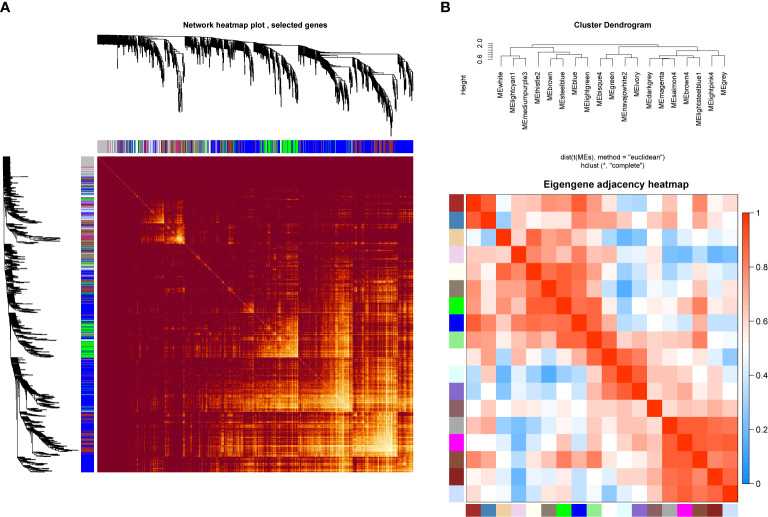
Network heatmap plot and clustered correlation. **(A)** The degree of correlation within the module. Different colors of horizontal and vertical axes represent different modules. The bright yellow in the middle of the plot represents the degree of connectivity between different modules. There was no significant difference in the interactions among the different modules, indicating a high-scale independence degree among these modules. **(B)** Eigengene adjacency heatmap.

In order to investigate the association between the pharmaceutical effect of TCM fractions and module eigengene (ME), the construction of weighted co-expressed networks and identification of co-expression modules were built through the “WGCNA” package in R. The sample dendrogram and trait heatmap are shown in **Figure 6A**. In this research, the power of β = 8 was set to guarantee high scale independence and low mean connectivity (**Figure 6B**). The dissimilarity of the modules was set as 0.2, and a total of 18 modules were generated (**Figure 7A**). The module trait relationship is shown in **Figure 7B**, and groups 2, 3, and 5 exhibited a higher connectivity in several modules. The blue and thistle2 modules associated with the cancer staging were the deepest, which suggest that these modules are suitable to identify the hub genes associated with the staging of cancer. The interaction relationships of the 18 modules are shown in **Figure 8A**. Each module was independent of each other, indicating a high-scale independence and differential genes expressions between the modules. The eigengene adjacency heatmap is shown in **Figure 8B**, the 18 modules were mainly divided into four main clusters.

### KEGG Pathway Enrichment Analysis of Blue and Thistle2 Modules

**Figure 9:**
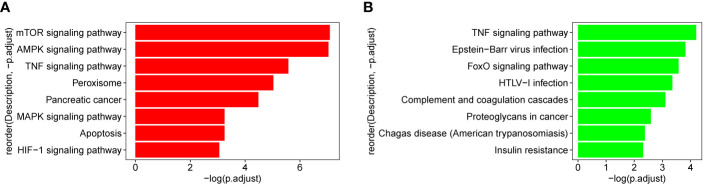
KEGG pathway of blue and thistle2 modules. **(A)** blue module comprises both the TNF and MAPK signaling pathways. **(B)** thistle2 module comprises the TNF signaling pathway.

The genes which are contained in each of the 18 modules were extracted and the KEGG pathway was constructed. As shown in **Figure 9**, genes from blue and thistle2 modules were mainly enriched on pathways involved in cell growth, infection, disease, and tumorigenesis. Interestingly, two candidate pathways were included, TNF and MAPK signaling pathways associated with the inflammatory processes in cancer.

### Hub Gene Identification in the Selected Modules

**Figure 10:**
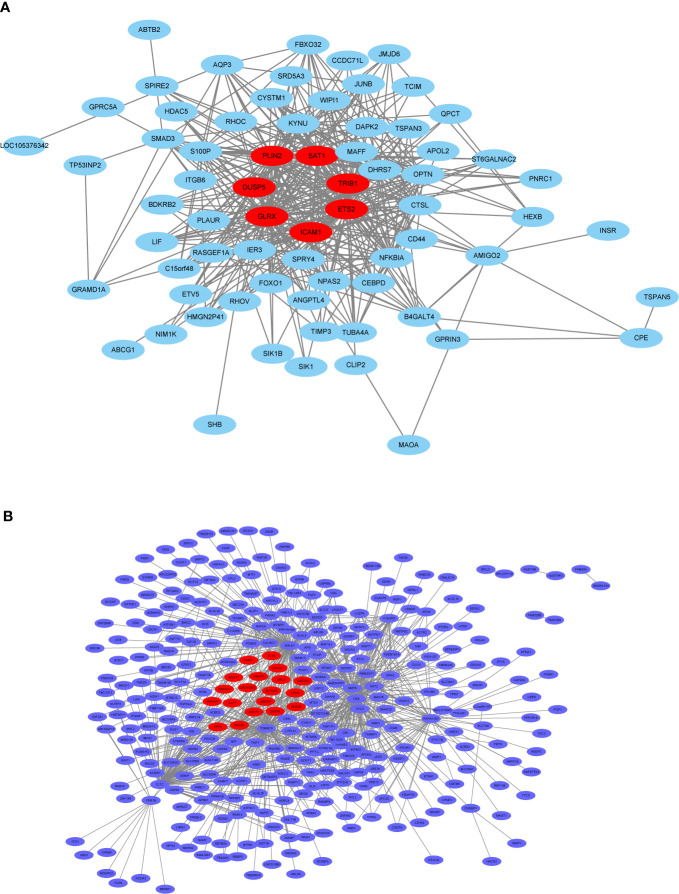
The co-expression network. **(A)** The co-expression network of the significant genes in the thistle2 module, including 72 nodes. **(B)** The co-expression network of the significant genes in the blue module, including 728 nodes. The hub genes were represented in red color. Cytoscape (v3.8.1) software was downloaded from the website (https://cytoscape.org/) and three models were chosen including “source node”, “target node”, and “edge attribute” with default parameters.

In general, genes included in the co-expression modules and with high connectivity are selected as hub genes. In this study, 26 hub genes (7 in thistle2 module and 19 in blue module) were obtained (**Figure 10**, **Supplemental Table 7**). The edges signifying the correlations in the thistle2 module was filtered by the criteria with a weight value > 0.035, then a total of 72 nodes were identified after importing to Cytoscape (v3.8.1) (**Figure 10A**, **Supplemental Table 7**). Meanwhile, blue module was filtered by a condition of the weight value > 0.24, 728 nodes were identified (**Figure 10B**, **Supplemental Table 7**).

## Discussion

Breast cancer is one of the leading causes of mortality among women in developing countries, and among other cancer, breast cancer has been ranked the highest, with an incidence of 25.8 per 100,000 and a mortality rate of 12.7 per 100,000 women (65, 77, 78). The main treatment methods for breast cancer include surgical resection, radiotherapy, and chemotherapy, however, these conventional therapies produce serious adverse effects, and chemotherapy drugs severely affect the function of the immune system. Currently, immunotherapy and targeted therapy have been widely used to destroy cancer cells (79). However, the clear information on biomarkers, target genes, effective components, key pathways, and interaction network of differential genes involved in tumorigenesis is required to support the treatment. Utilization of molecular networks as drug targets has been proved to be a next generation treatment for various cancers (80). On the other hand, the use of TCM has been reported as effective and healthy for cancer therapy and that it relieves adverse effects and enhances the efficacy of drugs. Moreover, the combination of modern medical methods and TCM in the process of tumor treatment significantly reduces the toxicity and side effects of radiotherapy and chemotherapy, decreases surgical complications and prolongs the survival of patients, and effectively improves their life quality (3, 9). In our study, we applied differential expression and WGCNA analysis and estimated the regulatory effect of three TCM candidates on the genes of five signaling pathways associated with biological processes including pro-inflammatory effects and metastasis process of breast cancer.

### The Chemical Components of Plant Samples

This study selected 74 TCM plant samples to screen their anti-cancer activity on breast cancer cell lines by high-throughput sequencing analysis. Some of them were found to have various chemical components regulating important pathways of breast cancer. For example, terpenoids can inhibit MCF-7 cell proliferation, promote apoptosis, and regulate several signaling pathways, including EMT, PTEN/PI3K/AKT, NF-kappa B, and Wnt/β-cathenin (21). Moreover, the flavonoids possess regulatory functions similar to terpenoids (21).The plant samples such as *E. cochinchinensis* Lour., *B. fruticosa*, *P. ginseng C. A. Meyer*, *E. hirta*, *P. japonica var major*, *F. suspensa*, and *S. scandens* were found to have anti-cancer compounds, terpenoids, and flavonoids. Alkaloids, especially the steroidal alkaloids, can perturb hedgehog signaling pathway and inhibit EMT in breast cancer cells similar to terpenoids and flavonoids (21). Bioactive alkaloids have been screened in various plants including *G. procumbens*, *T. divaricate*, *S. scandens*, *T. jasminoides*, *A. tagala*, and *C. aurantium L*.

Some of the plant samples used in this study, including *E. cochinchinensis Lour*., *B. fruticosa*, *E. hirta*, *P. reticulatus Poir.* var. *glaberMuell.-Age*., *P. massoniana Lamb*., and *P. aviculare*, were found to have the anti-cancer compound, Epigallocatechin-3-gallate (EGCG), which regulates EMT and CSCs in several estrogen receptors (ER)-negative breast cancer cells by downregulating ER-α36 expression (21, 81). Quercetin is an important flavanol, its combination with geldanamycin further potentiates the anti-proliferative and anti-migration effect of geldanamycin, enhances its inhibitory effects on ALDH+ cells, and controls mammosphere formation (24). Nine TCM candidates including *P. notoginseng*, *G. procumbens*, *S. scandens*, and *P. aviculare* have been found to have quercetin as a major active compound. Manassantin and arctigenin are plant lignans with anti-cancer activities, and regulates multiple important signaling pathways, including NF-kappa B, PI3K/Akt/mTOR, and MAPK/ERK, Notch-1, and p38. The plants *F. suspensa*, *S.* chinensi,s and *K. coccinea* were found to contain Arctigenin, which suppresses the phosphorylation of ERK 1/2 and JNK 1/2 in MCF-7 cells and thereby inhibits the MAPK signaling pathway. While, Manassantin is isolated from *F. suspensa*, *S. scandens*, *T. jasminoides*, and *Arctium lappa* (82).

Additionally, some of our TCM contain various chemical components associated with breast cancer treatment, it indicated that we should consider them as the key medicines for further investigation. *E. cochinchinensis Lour*. and *B. fruticosa* contain terpenoid, flavonoid, EGCG, and ursolic acid. *G. procumbens* contains flavonoid, oleanolic acid, quercetin, and quininic acid. Terpenoid, flavonoid, arctigenin, and lignan are included in *F. suspensa*. *S. scandens* contains terpenoid, quercetin, oleanolic acid, and hyperin. In *E. hirta*, terpenoid, flavonoid, and EGCG are included. *P. reticulatus Poir.* var. *glaberMuell.-Age*. contains terpenoid, flavonoid, and EGCG. Based on all the information and analysis results, we screened three candidate TCM to further investigate the medicinal compositions, pathways, and genes.

### Therapeutic Activities of the Three Candidate TCM

The leaves, roots, oils, fruits, and beans of *Ricinus communis L*. (*R. communis L*.) possess various therapeutic properties such as hepatoprotective, anti-inflammatory, diuretic, anti-cancer, anti-bacterial, insecticidal, hypoglycemic, and free radical scavenging activities (83). In particular, the fruit extract of this plant has been reported to have a potent anti-cancer activity (10, 16). Furthermore, the same fruit extract showed an anti-proliferative activity by inhibiting the migration, adhesion, and invasion of MCF-7 and MDA-MB-231 cell lines. It also induced apoptosis by reducing anti-apoptotic Bcl-2, inducing pro-apoptotic Bax expression and DNA fragmentation, as well as inhibiting upstream STAT3 activation responsible for the induction of MMPs and Bcl-2 (10). Bean extract has been recommended to use in the development of anti-inflammatory, anthelmintic, anti-bacterial, laxative, and abortifacient medicines. Meanwhile, Ricin is a main compound of *R. communis L*., that has been used to develop immunotoxins for killing the elimination of cancer cells (16). In addition, four probable compounds, including Ricinin, p-Coumaric acid, Epigallocatechin, and Ricinoleic acid, all showed anti-cancer activities (10).

*Excoecaria cochinchinensis Lour.* (*E. cochinchinensis Lour* Euphorbiaceae), namely “Hong-bei-gui-hua”, is widely distributed in the South and Southwest of China and cultivated largely as a green-tree, which has been used in traditional folk medicine to treat cancer, malaria, dysentery, furuncles, pruritus, prolonged diarrhea, and urethrorrhagia (84–86). It includes various compounds, such as highly oxygenated diterpenoids(named excolabdone D), loliolide, megastigmane glucosides, flavonoids, triterpenoids, sterols, and phenolic (17, 84). Among them, only (+)-epiloliolide was evaluated for its bioactivity. The preliminary pharmacological studies indicated that the EtOH extract showed cytotoxic activity against human Hela cell lines at a concentration of 12.5 ug/mL, while the MeOH extract of *E. cochinchinensis Lour.* leaves showed significant *in vitro* anti-inflammatory effects (18, 85, 86). Furthermore, a bio-assay guided study revealed the strong cytotoxic activity of the EtOAc fraction of this plant. In addition, betulinic acid and prostratin (a nontumor-promoting 12-deoxytigliane diterpenoid) have been shown, respectively, to inhibit HIV-1 replication and HIV expression in latently infected cell lines (86).

*Breynia fruticose* (*B. fruticose*, Euphorbiaceae), which is widely distributed in South China, is a folk medicine to treat gastroenteritis, sore throat, chronic bronchitis, wounds, and has an anti-cancer effect (19, 87). Previous research reported that *B. fruticose* contains almost 10 bioactive compounds. Among them, zizyberanalic acid and isoceanothic acid possess strong cytotoxic activity against five human cancer cell lines, including breast cancer MCF-7 (19). Meanwhile, phytochemical studies on *B. fruticosa* have revealed that its main active constituents include tannins, triterpenes, sterol derivatives, and lignins. The anti-inflammatory ingredients of *B. fruticosa* are sulfur containing sesquiterpenoids (breynins), which have strong anti-arthritic, anti-inflammatory effects of *B. fruticosa*. Some compounds similar with breynins, like thiacremonone, reduces inflammation by inhibiting the NF-κB DNA binding activity and expression of iNOS and COX-2, breynins may possess the similar mechanism (20). Furthermore, the chloroplast genome of *B. fruticosa* has been reported firstly, which can provide genomic resources of Breynia species for further research (87).

### Analysis of KEGG Pathway Enrichment in Different Treatments

KEGG analysis results are shown in **Supplemental Table 6**, the genes of some important pathways involved in cell cycle, carcinogenesis, pro-inflammatory effect, cancer metastasis breast cancer, small cell lung cancer (SCLC), prostate cancer, and bladder cancer were significantly enriched. The extracts of *E. cochinchinensis Lour*., *B. fruticosa*, *R. communis* L., *S. scandens*, and *M. verticillata* var. *crispa* significantly enriched the genes of the five pathways (TNF/IL-17/MAPK/NF-kappa B signaling pathway, Transcriptional misregulation in cancer). *M. azedarach* extract enriched the genes of the TNF and IL-17 signaling pathways, while the genes of the TNF and MAPK signaling pathways were enriched in cell lines treated with *M. barbatus* extract. *G. elegans* enriched only the MAPK signaling pathway. Interestingly, the extracts from the plants belonging to the Euphorbiaceae family significantly enriched the genes of all the pathways more than those from the plants of other families. *E. cochinchinensis Lour*., *B. fruticosa*, and *R. communis L*. *M. barbatus* enriched the genes of several important pathways and most of them were found to have active compounds like terpenoids, flavonoids, Epigallocatechin-3-gallate (EGCG), ursolic acid, Phenols, amino acids, Tannins, and Steroids. Terpenoids, flavonoids, and EGCG were found to be effective in breast cancer treatment (82). Ricinin, p-Coumaric acid, Epigallocatechin, and Ricinoleic acid extracted from *R. communis L*. were found to possess anti-cancer properties (10). Moreover, some plants of the Euphorbiaceae family such as *E. bicolor*, *E. hirta*, and *S. Baill* were analyzed for anti-cancer activity (88–90). In the positive controls, doxorubicin hydrochloride enriched the genes involved in the Cell cycle, p53 signaling pathway, and multiple cancer pathways, including breast cancer, non-small cell lung cancer, small cell lung cancer, bladder, gastric, pancreatic, and prostate cancer. It suggested that doxorubicin hydrochloride can be used as a broad-spectrum anti-cancer drug. Gemcitabine enriched the genes of the PI3K-Akt and FoxO signaling pathways. However, other positive controls enriched no cancer pathways, even a total of 28 samples enriched no pathways, they may weakly impact the MCF-7 cell.

### Enriched Pathways and Their Functional Association With Immunity and Progression of Cancer

Tumor Necrosis Factor alpha (TNF-a, TNF) is a key mediator and regulator, which regulates immune system development, cell survival signaling pathways, proliferation, and regulates metabolic processes. TNF-α is one of the essential pro-inflammatory cytokines of breast cancer patients found in the tumor microenvironment (TME), being secreted both by stromal cells (mainly tumor-associated macrophages) and the cancer cells themselves (70). TNF can also stimulate apoptosis in certain pathological conditions. Impacting the cell fate and organismal homeostasis is the main way of TNF-regulated pathways to participate in diverse cellular and physiological processes (70, 71).

The interleukin-17 (IL-17) family of cytokines is deeply implicated in chronic inflammatory diseases and is gaining interest as an actor in cancer immunity. IL-17 signaling is associated with immunopathology, autoimmune disease, and cancer progression (67, 75). Its pro-tumoral effects on proliferation, angiogenesis, invasion, migration, and resistance to treatments have been observed in a variety of cell lines and mouse models. These effects may be direct *via* widely measured IL-17Rs signaling through MAPK and NF-kappa B recruitment (67), IL-17 signaling pathway is primarily activated by serine/threonine kinases which characterize the NF-kappa B and MAPK pathway activation (75). NF-kappa B as a pro-inflammatory factor, activated by the IL-17-family in innate immune signaling. Numerous studies have shown that IL-17A activates various MAPKs, and that the MAPK pathway has an important role in regulating the expression of IL-17A-induced genes through the control of mRNA transcript stability (73–75).

The nuclear factor-kappa B (NF-kappa B, NF-κB) complex is composed of a family of inducible transcription factors found in almost all cells. Activating NF-kappa B would enhance the expression of several inflammatory cytokine genes, including TNF-α, IL-6, and IL-8 (72). NF-kappa B has been proved to participate in the initiation and progression of inflammation tumor tissues (91), and has even been regarded as a key coordinator of inflammation and important endogenous tumor promoter. NF-kappa B is involved in tumor cell growth and activated relative inflammatory cytokines, adhesion molecules, and angiogenic factors to expedite the proliferation of tumor cells (92, 93). NF-kappa B pathway is one of the main inflammation-mediated pathways, which may lead to the emergence of resistance to chemotherapy and endocrine therapy *via* the evasion of appropriate apoptosis (66).

Mitogen activated protein kinase (MAPK) pathways, which control proliferation, differentiation, and apoptosis and are also aberrantly regulated in cancer (94), are considered to play a crucial role in cellular signal transduction pathways. MAPK deregulation can lead to various cancer forms including breast, oral, lungs, colorectal, ovarian, and thyroid (65). In particular, it is an important signaling pathway associated with breast cancer progression (95). Targeting MAPK pathways along with conventional anti-cancer drugs enhances the effectiveness of drugs (96).

Transcriptional regulation is an important biological process of the cell and organism respond to multiple signals over their lifetime (97). Similarly, it is the core process in cancer progression and metastasis. Transcription factors (TFs) are the key regulators of gene transcription, and are often amplified, deleted, or rearranged to the gain or loss of function in a cancer cell (76). Therefore, transcriptional misregulation has a strong impact on the cancer metastasis process, such as local invasion, dissemination, and eventual colonization of the tumor to distant organs (69). Transcriptional misregulation also interacted with other pathways, for example, misregulation of the immune response transcriptional regulator NF-kappa B has been linked to inflammation in cancer, the transcription factor-controlled genes are involved in inflammation and was chronically active in cancer inflammatory process (76).

### The Common Genes of Five Pathways in Cancer Cells Treated With Three TCM Candidates

Pleiotropic cytokine IL-6 gene of the three key pathways (TNF, IL 17, and NF kappa B signaling pathways) was significantly upregulated in each of the treatments. It has been reported to be a key player in systemic inflammation and a biomarker for tumor burden and physical inactivity, since it possess the function of regulating cell growth, migration and renewal of stem cells, inflammation, and tissue metabolism (98). Similarly, IL-6 cytokine leukemia inhibitory factor (LIF), which belongs to the IL-6 family of cytokines, was recently identified as a critical factor in cancer progression, metastasis, stem cell maintenance and therapy resistance, and represented as a breast tumor and lung metastasis suppressor (99, 100). In addition, it has been reported that PIK3R1 and LYN regulate the PI3K/AKT signaling pathway in MCF-7 cell. PIK3R1 is a critical component of the PI3K/AKT signaling pathway, deregulation of the phosphoinositide 3-kinase (PI3K) pathway contributes to tumor development and progression. LYN is a member of the SRC family of protein tyrosine kinases (SFKs), which are key regulators of several cellular processes, including cancer cell growth, migration, invasion, and survival (101, 102). TNFAIP3 (The TNF-α induced protein 3), a potent anti-inflammatory and NF-kappa B inhibitory protein, protects cells from TNF-induced cytotoxicity. TNFα signals activate the NF-kappa B pathway *via* TNFR1 and TNFR2 in response to inflammation. Moreover, TNFAIP3 has been reported to control NF-kappa B activity and promote liver regeneration by activating the inflammatory IL-6/STAT3 signaling pathway. Its pro-proliferative mechanisms are increased and IL-6 induced STAT3 phosphorylation are sustained, decreasing the hepatocyte expression of the negative regulator of IL-6 signaling (103, 104). Peroxisome-proliferator activated receptors (PPARs) are related to the dysfunction of the skeletal muscle in women with breast cancer. PPARG (PPAR-gamma) is an important gene responsible for mitochondrial functions. Thus, PPARG signaling is crucial for breast cancer-derived factors altering lipid accumulation and mitochondrial functions (105). CCAAT/enhancer-binding protein beta (CEBPB) has been identified as a novel transcriptional regulator of the CLDN4 promoter. PAK4-mediated CEBPB activation upregulates CLDN4 expression to promote cell migration and invasion in breast cancer, thus, PAK4/CEBPB/CLDN4 may be a potential therapeutic target in breast cancer treatment. Furthermore, CEBPB also interacts with FOXO1, NF-kappa B, and CCL20, and FOXO1/CEBPB/NF-kappa B/CCL20 axis has been reported as a target for Colorectal cancer treatment (106, 107). Ephrin type-A receptor 2 (EPHA2) is a receptor tyrosine kinase (RTK), the overexpression of EPHA2 is associated with metastasis in a variety of cancers, including melanomas and ovarian, prostate, lung, and breast cancers. EphA2 receptor has a pro-oncogenic property which activates the tumor-suppressive signaling pathways of EphA2, and inhibits the PI3K/Akt and ERK pathways (108, 109).

Meanwhile, seven common genes, FOSL1, S100A9, CXCL12, ID2, PRS6KA3, AREG, and DUSP6, have been used as the target biomarkers and even the diagnostic tools in cancer therapy. FOSL1 is a basic-leucine zipper transcription factor which controls cell proliferation, differentiation, angiogenesis, cell mobility, and apoptosis. It plays a critical role in cancer cell invasion and metastasis. Overexpression of this gene has frequently been analyzed in multiple types of human cancers, including invasive breast cancers. High FOSL1 expression in TNBC patients leads to poorer survival rates than low FOSL1 expression (110, 111). S100A9 is a member of the S100 protein which shows a potential cytokine-like function in inflammation and is expressed in some cancers, impacting carcinogenesis, cancer development, and metastasis. It has been reported that S100A9 probably plays a key role in inflammation-related breast cancers and induces the expression of pro-inflammatory cytokines (IL-6, IL-8, and IL-1β), thus, its expression level can be used to diagnose breast cancer (112, 113). The chemokine CXCL12 has been shown by therapies against aggressive and metastatic breast cancer to regulate the cell growth and metastasis of breast cancer, and to develop the tumor microenvironment. It can also be used as a cancer biomarker and adds prognostic information in various cancer types (114, 115). Inhibitors of DNA-binding (ID) proteins contribute to cell proliferation and differentiation. The member ID2 has been reported as a prognostic marker in breast cancer patients, as well as a key regulator of breast cancer metastasis to the brain (116, 117). RPS6KA3 (RSK2). Moreover, it has also been proposed as a target for cancer therapy due to it working as a transcription factor in breast cancer, especially, in ER- breast cancer (118). Amphiregulin (AREG) is an epidermal growth factor (EGF) receptor ligand found in multiple cancer types. AREG is co-expressed with AR in invasive breast cancer, which is reported as one of the prognostic biomarkers and therapeutic targets in invasive breast cancer, particularly in ER-negative breast cancer (MCF-7). EGFR‐mediated Akt activation is associated with PD‐L1 expression, which can be reduced by EGFR inhibitors in cancer cell lines carrying activated EGFR (119, 120). DUSP6 is a member of the MAPK phosphatase family which plays a pro-oncogenic role in human glioblastoma, thyroid carcinoma, breast cancer, and acute myeloid carcinoma *via* the MAPK signaling pathway. It has also been reported as a potential therapeutic target and diagnosis marker for different cancers (121).

Based on these results, future research could probe the interaction between medicinal plant ingredients and breast cancer cells, and to explore the differential gene activation mechanisms for pharmacodynamic substances to identify their medicinal compositions and action mechanisms (2, 4). Furthermore, we hope to identify novel makers, screen out breast cancer therapeutic targets, and develop new natural drug groups against breast cancer, bringing new ideas to the development of new drugs screening methods (31, 33, 34).

## Data Availability Statement

Publicly available datasets were analyzed in this study. This data can be found here: Sequence Read Archive (SRA) database of the National Center for Biotechnology Information (NCBI). The BioProject number is PRJN718482, SRA experiments number is SUB8681695 (https://dataview.ncbi.nlm.nih.gov/object/PRJNA718482?reviewer=ggtl9pevdaidn4a08lg76b8p1c).

## Author Contributions

JM and QK conceived this study. XNY, KW, XZ, SW, and XZY performed the experiments. LK, YP, and ZX performed the bioinformatics analyses. LK wrote the manuscript. JC, YL, and AM edited the manuscript. All authors contributed to the article and approved the submitted version.

## Funding

This work was supported by the grants from the Key Project of TCM Modernization Research (2019YFC1711000), Jiangsu University (No. 20JDG47), Guangxi Science and Technology Research Project (GuiKeAA18242040, GuiKeAD17129044).

## Conflict of Interest

The authors declare that the research was conducted in the absence of any commercial or financial relationships that could be construed as a potential conflict of interest.

## Publisher’s Note

All claims expressed in this article are solely those of the authors and do not necessarily represent those of their affiliated organizations, or those of the publisher, the editors and the reviewers. Any product that may be evaluated in this article, or claim that may be made by its manufacturer, is not guaranteed or endorsed by the publisher.
